# An In-Person and Telemedicine “Hybrid” System to Improve Cross-Border Critical Care in COVID-19

**DOI:** 10.5334/aogh.3108

**Published:** 2021-01-04

**Authors:** Venktesh R. Ramnath, Linda Hill, Jim Schultz, Jess Mandel, Andres Smith, Tim Morris, Stacy Holberg, Lucy E. Horton, Atul Malhotra, Lawrence S. Friedman

**Affiliations:** 1 Division of Pulmonary, Critical Care, and Sleep Medicine, UC San Diego Health, La Jolla, CA, US; 2 Department of Family Medicine and Public Health, UC San Diego Health, La Jolla, CA, US; 3 Neighborhood Healthcare, San Diego, CA, US; 4 Department of Emergency Medicine, Sharp Healthcare, San Diego, CA, US; 5 Director, International Program Operations, UC San Diego Health, La Jolla, CA, US; 6 Division of Infectious Diseases, UC San Diego Health, La Jolla, CA, US; 7 Department of Internal Medicine, UC San Diego Health, La Jolla, CA, US

## Abstract

**Background:**

UC San Diego Health System (UCSDHS) is an academic medical center and integrated care network in the US-Mexico border area of California contiguous to the Mexican Northern Baja region. The COVID-19 pandemic deeply influenced UCSDHS activities as new public health challenges increasingly related to high population density, cross-border traffic, economic disparities, and interconnectedness between cross-border communities, which accelerated development of clinical collaborations between UCSDHS and several border community hospitals – one in the US, two in Mexico – as high volumes of severely ill patients overwhelmed hospitals.

**Objective:**

We describe the development, implementation, feasibility, and acceptance of a novel critical care support program in three community hospitals along the US-Mexico border.

**Methods:**

We created and instituted a hybrid critical care program involving: 1) in-person activities to perform needs assessments of equipment and supplies and hands-on training and education, and 2) creation of a telemedicine-based (Tele-ICU) service for direct patient management and/or consultative, education-based experiences. We collected performance metrics surrounding adherence to evidence-based practices and staff perceptions of critical care delivery.

**Findings:**

In-person intervention phase identified and filled gaps in equipment and supplies, and Tele-ICU program promoted adherence to evidence-based practices and improved staff confidence in caring for critically ill COVID-19 patients at each hospital.

**Conclusion:**

A collaborative, hybrid critical care program across academic and community centers is feasible and effective to address cross-cultural public health emergencies.

## INTRODUCTION

The UCSD Health System (UCSDHS) is an academic medical center with an integrated care network located at one of the most populous sections of the US-Mexico border area. UCSDHS is situated in a section of this border area with two sister-city regions, San Diego-Tijuana and El Centro-Mexicali, over a distance of roughly 110 miles from west to east.

## PUBLIC HEALTH AND THE BORDER REGION OF THE US

The public health importance of this border area is well recognized given its population density and social, economic, and political importance [1]. The interdependence of these cross-border regions is characterized by high traffic across the US-Mexico border, particularly at the San Ysidro Land Port of Entry (the busiest border crossing in the Western Hemisphere) [2], as US citizens and legal residents reside in Mexico for lower costs of living but maintain employment in the US. Legal border crossings at the San Ysidro and Otay Mesa border normally exceed 130,000 daily and initially decreased to 40,000 daily with governmental COVID-19 travel restrictions in March 2020 but eventually returned to 80,000 daily by June 2020 [3]. During this time, more than 90% of crossings were by US citizens or legal permanent residents of the US. As noted by the US-Mexico Border Health Commission: “…it is important to understand the border region as a microcosm where bilateral relations and prevailing implications are considered when developing targeted solutions to address public health challenges.” [1]

## COVID-19 AND THE URGENCY FOR MULTI-INSTITUTIONAL COLLABORATION

**Figure 1:**
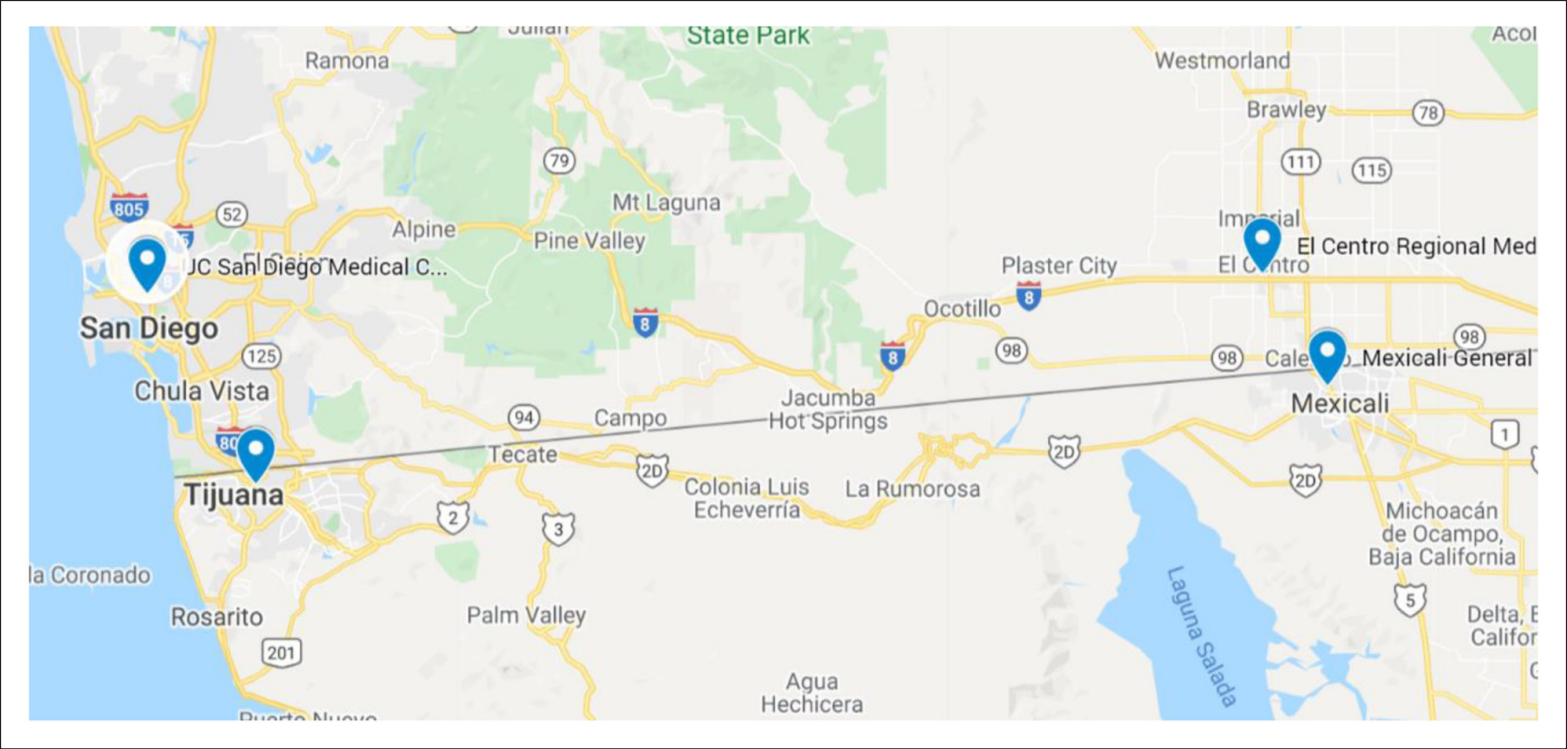
Location of El Centro Regional Medical Center (El Centro, CA), Tijuana General Hospital (Tijuana, MX), and Mexicali General Hospital (Mexicali, MX), in relation to US-Mexico border and UCSDHS, located in San Diego, CA [29].

In March 2020, southern California and the Baja region of Mexico began experiencing case surges of severe COVID-19 illness (rapidly progressive respiratory and multi-system organ failure due to SARS-CoV2 viral infection requiring critical care support, including invasive mechanical ventilation) [4] that quickly saturated hospitals serving these sister-city regions, resulting in a domino effect, as Mexican hospitals looked to US border hospitals for help, and US border hospitals looked to other US hospitals for assistance. The geographic proximity between the sister-city hospitals, as well as to UCSDHS, encouraged the development of a collaborative program (see Figure 1). Ultimately, regional healthcare leaders from the US-Mexico border reached out to San Diego County Public Health Services and UCSDHS for guidance.

While the Baja region of Mexico did not officially report high rates of COVID-19 until mid-to-late April (approximately one month after SD), public health measures implemented in SD County alone quickly proved inadequate to contain the regional needs across the Mexican border and in Imperial County in California. Furthermore, public health forecasts and models indicated that cases would escalate well into the summer months of 2020 and that the large US expatriate population (~250,000 in Baja California and Baja Sur, ~75,000 of those in Mexicali) would seek care not only locally in Mexico but also across the border in the US. True to predictions, the regions of SD and Imperial Counties still continue as some of the “hottest” for COVID-19 infections in the US) [5,6].

Given longstanding missions that focus primarily on tertiary clinical care, teaching, and research, academic health centers may sometimes be viewed as out-of-step with the needs of community health [7,8]. However, most agree that healthcare delivery for all US citizens and residents involves a basic Hippocratic imperative that obligates healthcare providers to intervene strategically for those at highest risk and whose condition also may affect public health and safety. As a result, a call to academic centers has been made during this pandemic to take decisive action to advance regional and national health and mitigate healthcare disparities [9]. Upon this principle, UCSDHS engaged to address the COVID-19 pandemic in our border region.

## TELEMEDICINE AND CROSS-CULTURAL CONTEXTS

While telemedicine has shown utility in developing nations and other underserved contexts, technologies vary widely and largely involve non-critical care patients [10]. Furthermore, despite augmented global interest in telehealth during the COVID-19 pandemic, there are notable barriers to its adoption and implementation [11].

Existing telemedicine-based critical care solutions (“Tele-ICU”) are differentiated into centralized and decentralized systems [12,13] based on the presence or absence of a remotely located “hub” of ICU personnel that receives patient data and directs care management. Standalone Tele-ICU systems (centralized and decentralized) have improved access to critical care in underserved populations but with mixed efficacy due to variations in technology platform, study design, and patient characteristics [14]. We created a hybrid program for the border hospitals utilizing both in-person support and a Tele-ICU platform. An independent, direct care-oriented Tele-ICU clinical service line was launched in the US-located hospital (ECRMC), while a case-conference-styled, educationally-focused Tele-ICU model was used in the Mexican hospitals (HGT, HGM) (see Table 1).

**Table 1:** Description of Tele-ICU intervention at each border hospital site. HGM, Mexicali General Hospital (Mexicali, MX); HGT, Tijuana General Hospital (Tijuana, MX); ECRMC, El Centro Regional Medical Center (El Centro, CA, USA). RN = Registered Nurse; RT = Respiratory Therapist


TYPE OF TELE-ICU INTERVENTION	HGM	HGT	ECRMC

Direct management (order-writing, note-writing, care plan review with RNs, RTs, hospitalists, etc.)			✓

Consultative/educational (individual case presentation and discussion of active patients, with specific recommendations given to bedside providers only) and/or review of relevant journal articles on severe COVID-19 patient management	✓	✓	Only upon request by bedside providers (occurred approximately once per month)


We detail our interventions and outcomes to date (through July 2020) as we enacted a hybrid in-person/Tele-ICU service to address ICU needs of several US-Mexico border hospitals – one US hospital (ECRMC), located in El Centro and two Mexican municipal hospitals, located in Tijuana (HGT) and Mexicali (HGM).

## INTERVENTION

We strategized a hybrid, two-phase intervention based on our prior success in launching community-based critical care outreach programs elsewhere in the UCSDHS network.

### IN-PERSON APPROACH

After formal site visits to each hospital by members of the UCSDHS-led team (HGT/HGM: J.M., L.C., L.H. J.S., A.S., K.O.; ECRMC: V.R., K.A.), an assessment of ICU infrastructure, personnel, and supplies (see Appendix 3), clarification of the role for subsequent Tele-ICU involvement, and a detailed site-specific in-person intervention strategy were made. At HGT, a team of one critical care physician, multiple critical care nurses, a respiratory therapist, and a certified medical interpreter attended bedside ICU rounds and provided individualized clinical guidance daily for thirty days to help establish a foundation of knowledge and skills in ICU management. Activities ranged from development and dissemination of ICU algorithms and protocols to equipment recommendations (following direct review of inventories and equipment by UCSDHS staff). Nine semi-structured interviews (three in English, given high English fluency of the interviewees, and the remainder in Spanish with official translators) between hospital leaders, frontline ICU clinical providers, and UCSDHS staff addressed various domains: adequacy of processes of care and availability of equipment and perceived efficacy in caring for critically ill COVID-19 patients. At HGM, a team of ICU nurses, one intensivist, one infectious disease physician, and a medical interpreter spent seven days in Mexicali to provide on-site support similar to what was performed in HGT. At ECRMC, in-person services were only pursued later (July 2020) given financial constraints and earlier external support from the US Armed Forces and National Guard.

### TELEMEDICINE APPROACH

The Tele-ICU intervention phase followed in-person assessment at all sites. Our assessment indicated that a “decentralized” Tele-ICU system, in which tele-intensivists provide care from points of convenience (e.g. home, office, or via mobile devices), rather than from a centralized hub [15,16,17], would have the highest yield at all sites.

At ECRMC, Tele-ICU provided direct clinical management as critical care consultants upon request by hospitalists, who were the primary attendings of record in an “open” (low-intensity) staffing model ICU. Dedicated educational sessions and/or coaching exercises were done by request only (not formally included in the model). The ECRMC Tele-ICU service directly interacted with patients, family members, hospitalist physicians, other consultants, and nursing, respiratory therapy, and ancillary staff to create, execute, and coordinate care plans.

At HGM and HGT, in contrast, and in part due to medical licensure limitations, Tele-ICU provided case conference-style, education-based sessions with Mexican physicians only. In these one-hour sessions taking place two mornings each week, HGT/HGM physicians presented clinical information (lab/imaging data, medications and dosages, ventilator settings, etc.) from two to four ICU patients with severe COVID-19 illness (i.e. mechanically ventilated) and asked specific management questions for advice in each case. UCSDHS intensivist consultants provided direct diagnostic and therapeutic recommendations for each case, answered specific questions, and discussed pertinent medical journal articles as required or requested by physician staff. Unlike Project ECHO [18], which largely provided general approaches to COVID-19 ICU patient management in didactic tutorials, Tele-ICU in the Mexican hospitals provided direct, specific recommendations and advice on active individual cases.

Tele-ICU service at each site used two-way audio-visual telemedicine technology. At HGT/HGM, Tele-ICU sessions were scheduled at times to accommodate rounding schedules and shift changes, while ECRMC had a fixed schedule (8am to noon, daily). In all cases, the in-person activities served to establish a trust-based relationship founded on a commitment to common patient-oriented goals.

## EVALUATION OF IMPACT

Institutional Review Board approval was not sought, because this initiative was quality improvement related to COVID-19 and not research per se.

At ECRMC, we assessed effects of Tele-ICU through the following: 1) tele-intensivist physicians marked any specific areas of management (in which evidence-based practices were recommended due to observed deviations) on an electronic (Excel) spreadsheet shared through a secure, cloud-based server (see Appendix 1); 2) qualitative, structured surveys (see Appendix 2) were distributed by email via electronic survey link (Survey Monkey®) by V.R. to all nurses (30), hospitalist (7), and respiratory therapy (14) staff who cared for ICU patients exactly two months after Tele-ICU launch (ECRMC), addressing staff satisfaction with the service, confidence in care, and identification of areas for improvement. Spreadsheet audits were conducted daily by V.R. to ensure 100% compliance during the study period. Requests to staff to complete surveys was done upon distribution and follow-up three days after distribution, with all responses collected within one week of distribution. Results were blinded to response results but unblinded regarding number of specific staff within each group who responded (i.e. hospitalist physicians, nurses, respiratory therapists). All survey results were reviewed by V.R. and analyzed using application-based software (SurveyMonkey®).

At HGT/HGM, UCSDHS staff performed direct reviews of inventories and equipment and nine qualitative interviews with hospital leaders and frontline ICU staff in English and Spanish with official translators as required. Direct, quantitative measurement of impact in HGT and HGM was not possible for several reasons. Most important, the urgency of the need prioritized organization and implementation of existing resources over other considerations. As UCSDHS staff were volunteers, streamlining tasks was necessary, and our assistance was intended as a humanitarian and collegial gesture to improve quality rather than an investigational exercise. The need for basic equipment and staff numbers presented clear gaps in care delivery that made equipoise difficult. Finally, logistic challenges (e.g. WiFi strength, scheduling, etc.) and cultural differences (e.g. language barriers) made data collection more challenging.

## RESULTS

### PROGRAM SCOPE AND IMPLEMENTATION

At each site, a UCSDHS-led team successfully identified gaps in existing ICU delivery mechanisms, provided targeted recommendations, and launched a Tele-ICU service.

At HGT, specific recommendations included 1) purchase of equipment and supplies for mechanically ventilated patients (eg. active humidification systems, bedside oximetry and vital sign monitors) and 2) institution of management algorithms (eg. tidal volume selection, PEEP titration, ventilator liberation, enteral nutrition, sedation, etc.) and nursing protocols (eg. prone positioning). The HGT clinical teams welcomed these suggestions and invited UCSDHS-led teams for further visits and recommendations. In addition, a team of HGT physicians and nurses visited UCSDHS hospital campuses (Hillcrest and Jacobs Medical Centers) to observe care practices. The Tele-ICU service started on June 22, 2020 with 12 sessions through Aug 1, 2020, with an average of two patient cases discussed per session (24 tele-case conferences performed), during which ventilator management, sedation practices, thromboprophylaxis, fluid management, and antimicrobial use and indication were discussed.

At HGM, specific recommendations included enhanced nursing protocols, infection control strategies, and clinical management (e.g. sedation, ventilator management, fluid balance, etc.). The clinical teams incorporated the input internally. The UCSDHS team provided 10 days of in-person nursing educational support and seven days of in-person, physician-led educational support. The Tele-ICU service began on June 22, 2020 with 12 sessions through Aug 1, 2020, with one patient case discussion per session alternating with review of a peer-reviewed journal articles regarding COVID-19 management (eight tele-consultations and four articles reviewed). Case discussion topics were the same as those in HGT. Plans are in process for the HGM team to visit UCSDHS.

At ECRMC, Tele-ICU services launched on April 6, 2020, serving 169 unique patients through August 1, 2020. Since inception, the following increased: 1) number of coverage days requested (increasing from 10 days/month in April to daily by July 1, 2020), 2) number of total tele-encounters (48 in April 2020 to 188 in July), and 3) encounters per patient (suggesting improved continuity of care). The Tele-ICU service attended patients with severe COVID-19 almost exclusively (> 98%). Interventions to ensure evidence-based critical care grew after program launch, especially regarding advanced ventilator management, sedation management, and hemodynamic management, but intervention rates reduced in some cases by July 2020 (Table 2). While speculative, this finding may suggest early adoption of evidence-based practices that may lead to cultural change in critical care delivery. While we did not capture the actual adoption rate of recommendations, personal observations by staff suggested that they were followed almost 100% of the time. When not followed, clinical conflicts between tele-intensivists and hospitalists were cited.

**Table 2:** Frequency of interventions by the Tele-ICU service to achieve evidence-based standard of care in the management of critical care patients at ECRMC. More than one intervention within each category was seen in some cases


INTERVENTION	INTERVENTIONS MADE TO ACHIEVE STANDARD OF CARE (% OF TOTAL ENCOUNTERS)			

	APRIL 2020	MAY 2020	JUNE 2020	JULY 2020

Prone positioning	0	57	30	57

	(0%)	(46%)	(20%)	(30%)

Advanced ventilator management	18	75	194	211

	(38%)	(60%)	(134%)	(112%)

Hemodynamic management	2	20	50	50

	(4%)	(16%)	(34%)	(27%)

Fluid management	1	70	90	121

	(2%)	(56%)	(63%)	(64%)

Sedation/neuromuscular antagonist management	12	55	88	100

	(25%)	(44%)	(61%)	(53%)

Nutrition and bowel motility	0	6	90	79

	(0%)	(5%)	(63%)	(42%)

Venous thromboembolism prophylaxis	0	0	32	12

	(0%)	(0%)	(22%)	(6%)

Stress ulcer prophylaxis	0	0	28	21

	(0%)	(0%)	(19%)	(11%)


### SPECIFIC FEEDBACK ON THE INITIATIVE INCLUDING STAFF CONFIDENCE

**Figure 2:**
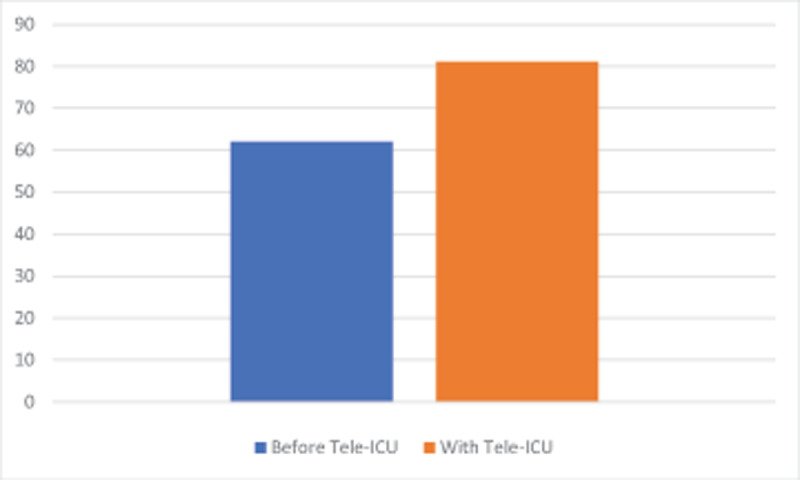
Percentage of ICU patients perceived to be receiving evidence-based care at ECRMC by respiratory therapy, nursing, and physician staff at ECRMC, as a result of Tele-ICU adoption. Before Tele-ICU refers to prior to April 6, 2020. Survey conducted on June 28, 2020.

**Figure 3:**
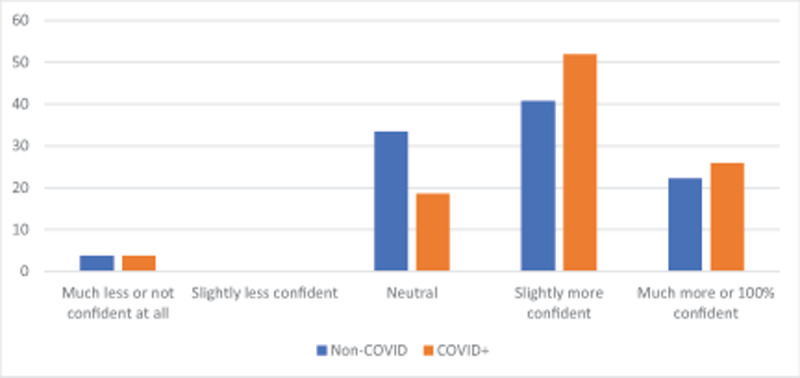
Change in confidence level of 7 physicians, 10 respiratory therapists, 10 nurses at ECRMC, as a result of Tele-ICU adoption. Note that confidence levels increased even in the care of patients without COVID-19 illness, who almost never were seen by Tele-ICU service, indicating a cross-over effect.

At ECRMC, of the 51 surveys sent out, 27 individuals returned responses (seven physicians, 10 respiratory therapists, and 10 nurses), corresponding to a response rate of 100% for physicians, 71% of respiratory therapists, and 33% of nurses (total response rate 53%). Nineteen percent more patients received evidence-based practice as a result of Tele-ICU (61.8% to 80.6%) (p < 0.0001 assuming paired t test; 2-tailed; standard deviation 25.3 vs 21.7), 78% of staff were more confident in caring for COVID-19 patients in the ICU (26% were definitely more confident or 100% confident), and 63% of staff were more confident (22% definitely more confident or 100% confident) in the care of non-COVID-19 ICU patients (see Figures 2 and 3). Highest value was attributed to enhanced care of mechanically ventilated patients (63%) and the ability to ask medical questions to an intensivist (19%). Regarding program improvement, 37% desired better consistency of plan between tele-intensivists and onsite physicians (eg. ECRMC hospitalists), while 72% requested more Tele-ICU coverage hours.

One-on-one unstructured interviews with hospital leaders (nursing, physician, and respiratory therapy) at all three hospitals demonstrated various qualitative gains of Tele-ICU service:

- Increased comfort and confidence with prone positioning in routine practice (ECRMC, HGT, HGM)
- Increased confidence in safely extubating patients (ECRMC)
- Enhanced targeting of specific areas for quality improvement (e.g. titration of neuromuscular blockers and dexmedetomidine in ventilated patients, concentration of drips for enhanced fluid management, enteral feeding-based nutrition goals, optimal timing of ancillary testing, etc.) (ECRMC, HGT, HGM)
- Improved access to advanced life-saving therapies (i.e. ECMO [extracorporeal membrane oxygenation]). Two patients even received onsite cannulation via a new “mobile ECMO” service at UCSDHS [19] (ECRMC)

## DISCUSSION

We describe our experience at UCSDHS as we responded to requests of one US and two Mexican hospitals in the US-Mexico border area seeking assistance with COVID-19 by deploying a hybrid program of in-person as well as telemedicine services. Our experience highlighted the following:

- **Collaborative public health programs in border regions are crucial for the health of both communities**.

The COVID-19 pandemic accentuated the public health implications of the San Diego-Imperial-Baja region, known for fluid movement between counties and countries. As commercial and non-commercial traffic continues to cross “closed” borders legally at high volume, essential workers in lower income social strata who live and travel frequently and in close proximity remain particularly prone to viral transmission and disease.

We found that institution of our program improved care such that quality of care – and perhaps more important, *perception* of care – was improved in the eyes of community healthcare providers. As our results showed that 78% of local healthcare providers felt more confident to care for critically ill COVID-19 and 63% were more confident with non-COVID patients, the UCSDHS intensivist program demonstrated putative impacts upon improved morale and work performance [20]. As community members themselves, local healthcare workers also contribute to increased public willingness to seek healthcare in nearby, rather than distant, hospitals and medical centers. Indeed, while data are still accruing, we expect that trends to date will continue, indicating that fewer patients from the border region require transfer to other US hospitals, thereby reducing displacement of patients from their families as well as burdens and costs upon transportation agencies and other hospitals.

- **An academic center can feasibly and rapidly create a program to support low-resource community hospitals during a pandemic or other medical crisis**.

Despite fears of bureaucratic hurdles and high costs of engaging academic services, we demonstrated both feasibility and quick implementation of a cost-effective program that won universal support. Contracts and agreements were created expeditiously (within days) despite the existence of public lockdowns, employee furloughs, and widespread stress during the pandemic. Of note, all involved staff (nurses, therapists, physicians, and interpreters) participated on a volunteer basis in the Mexican hospitals, such that longer sustainability will require expansion of the volunteer pool.

Others have indicated that use of implementation and team science is required for innovative change and process improvements in critical care [21,22,23,24], including relations building between academic medical centers and community ICUs [25]. However, few have described the experience of launching such a collaboration in a cross-border context with the unprecedented urgency of COVID-19 pandemic. While our experience is particularly relevant to health centers at US-Mexico border, academic and community centers can leverage collaborative in-person and telemedicine services in other cross-cultural challenges in critical care delivery (e.g. urban and rural contexts). Our experience demonstrates that partnerships are possible despite short timeframes and various operational stresses attributed to COVID-19 such as lockdowns, employee furloughs, and restricted travel.

- **Programs that combine in-person and telemedicine systems are likely synergistic to generate the best outcomes**.

Hybrid systems offer complementary advantages: in-person involvement allows for direct sharing of ideas, enhanced collegiality, and morale building while telemedicine (Tele-ICU) platforms leverage cost-effective technology. In addition, in-person visits permit direct observation of nursing, infection control, and other institutional practices that impact ICU outcomes but fall outside of traditional workflows. The time saved through virtual rounding – as opposed to traveling to sites in person – also allows broader participation of academic providers with competing responsibilities. Furthermore, these benefits appear synergistic, as stronger relationships between the remote and onsite providers lead to higher usage of telemedicine systems [26,27], which we believe are directly responsible for our positive results. At ECRMC, we expect further improvements as we have added an in-person, onsite intensivist service.

We found that awareness and implementation of evidence-based practices of critical care in COVID-19 [28] increased considerably over the course of the program, between launch in early April 2020 through June 2020. At ECRMC, interventions addressed adherence to recommendations regarding advanced ventilator management, vasopressor selection, sedative selection, election of specific COVID-19 specific agents, and recommended fluid and diuretic management.

Perhaps more important, the ECRMC Tele-ICU service increased staff confidence in COVID-19 care. While less pronounced, ICU staff also reported increased confidence regarding patient care for non-COVID-19 ICU patients (for whom the Tele-ICU service was not involved). One interpretation of these findings supports the presence of cultural osmosis, but further study is required.

In all three hospitals, our Tele-ICU case discussions led to idea sharing between academic and local hospitals that was fulfilling to both parties. The HGT and HGM hospital staff not only felt reassured that our recommendations generally matched their own clinical instincts, but also expressed interest in learning how to “fine-tune” their approach to advanced ventilator management (eg. assessment of “recruitability,” pleural pressure, transpulmonary gradient, prone positioning techniques/duration, etc.), use of sedatives and neuromuscular blockade, and other advanced ICU concepts. Concurrently, UCSDHS staff found the experience valuable, appreciating firsthand the strong knowledge base and experience of HGT/HGM practitioners and engaging in active discussions about medical evidence. Furthermore, UCSDHS tele-intensivists gained familiarity with challenges faced by seasoned practitioners managing patients with similarly high acuity of illness as in academic centers, but with severe resource limitations. In fact, UCSD intensivists experienced less stress in home campus ICU shifts due to enhanced appreciation of the efforts of community health providers and the involvement of UCSD colleagues in the program (personal observations).

In addition, our GoFundMe campaign raised ~$40,000 in funds successfully in June 2020 that were allocated to the purchase of ventilator parts (e.g. active humidification systems, oximeters, bedside monitors), and other equipment and supplies for HGT and HGM. Thus, we found that crowdfunding is an effective tool to address equipment and supply shortages in humanitarian settings, in addition to other strategies such as philanthropic and charity-based funding and donations. Keeping these strategies in mind for the longevity of our program itself, we are actively seeking philanthropic and foundation-based support for the continuation of our initiative.

## LIMITATIONS

There were several limitations to our experience and findings, primarily due to the rapidity of the efforts during this crisis. First, our data were purely observational and did not include robust study design, leading to various risks of bias when interpreting our results (e.g. selection bias, sampling bias, observer bias (non-blinding), immortal time bias, and confounding). Definitive conclusions about interventions require proper controls and epidemiologic study design. Second, our local results pertain to the US-Mexico border, such that generalizability of results is unclear. Third, each site (HGT, HGM, and ECRMC) had unique circumstances, needs, and historical relationships with UCSDHS that create challenges in comparing each experience to each other. Future evaluations are underway to create more standardized processes such that a more rigorous understanding of this initiative can provide more representative conclusions.

## CONCLUSION

We highlight our COVID-19 experience at UCSDHS regarding ICU regional needs at the US-Mexico border. We found that 1) public health approaches are important to address clinical concerns on both sides of the border, 2) an academic-community ICU partnership could be devised and executed in a short time frame under stressful pandemic conditions, and 3) implementation of a hybrid (in person and Tele-ICU) critical care program generated synergistic value. While the experience at each hospital was slightly different, common principles of team building, idea sharing, and adherence to evidence-based ICU practice were prominent. We feel that our experience can serve as a guide for other academic and community centers looking to build collaborative programs to address public health emergencies in cross-cultural contexts.

## ADDITIONAL FILES

The additional files for this article can be found as follows:

10.5334/aogh.3108.s1 Appendix 1. Data fields in the cloud-based spreadsheet for Tele-ICU provider interventions and assessments. Providers indicated all interventions ordered and assessments made for each patient each day. Responses were binary (Yes/No), except in the “Other,” which was free text field. PBW = predicted body weight; MAP = mean arterial pressure; RASS = Richmond Agitation Sedation Scale; TOF = Train of Four.

10.5334/aogh.3108.s2 Appendix 2. Survey questions as part of staff perception evaluation. RN = registered nurse; RT = respiratory therapist.

10.5334/aogh.3108.s3 Appendix 3. Assessed factors upon initial assessment. HGT = Tijuana General Hospital, HMG = Mexicali General Hospital, ECRMC = El Centro Regional Medical Center, HME = Heat and Moisture Exchangers, EMR = Electronic Medical Record, EUA = Emergency Use Authorization.

## References

[1] United States-Mexico Border Health Commision. Healthy Border 2020: A Prevention and Health Promotion Initiative 2020 https://www.hhs.gov/sites/default/files/res_2805.pdf. Accessed June 24, 2020.

[2] WorldAtlas. The Busiest Border Crossings in the United States 2019 https://www.worldatlas.com/articles/the-busiest-border-crossings-in-the-united-states.html. Accessed July 17, 2020.

[3] US Customs and Border Protection. Statistics on border crossings at San Ysidro and Otay Mesa ports of entry.

[4] Berlin DA, Gulick RM, Martinez FJ. Severe Covid-19. N Engl J Med. 2020 DOI: 10.1056/NEJMcp2009575 32412710

[5] Sieff K. Coronavirus on the Border: California Hospitals Overwhelmed by Patients from Mexico. The Washington Post. 2020 https://www.washingtonpost.com/world/2020/05/27/coronavirus-mexico-border/?arc404=true. Accessed June 25, 2020.

[6] Lah K, Jones J, Berryman K. Medics are Down to Their Last Defense with Coronavirus Swamping Their Town. CNN. 2020 https://www.cnn.com/2020/07/09/us/california-coronavirus-imperial-county/index.html. Accessed July 12, 2020.

[7] Newton WP. Shaping the future of academic health centers: The potential contributions of departments of family medicine. Ann Fam Med. 2006; 4(suppl_1): S2–S11. DOI: 10.1370/afm.587 17003157 PMC1578669

[8] Michener L, Cook J, Ahmed SM, Yonas MA, Coyne-Beasley T, Aguilar-Gaxiola S. Aligning the goals of community-engaged research: Why and how academic health centers can successfully engage with communities to improve health. Acad Med. 2012; 87(3): 285–291. DOI: 10.1097/ACM.0b013e3182441680 22373619 PMC3292771

[9] Awosogba T, Betancourt JR, Conyers FG, et al. Prioritizing health disparities in medical education to improve care: Prioritizing health disparities in medical education. Ann N Y Acad Sci. 2013; 1287(1): 17–30. DOI: 10.1111/nyas.12117 23659676 PMC4598316

[10] Wootton R, Patil NG, Scott RE, Ho K. Telehealth in the Developing World. IDRC; 2009.

[11] Smith AC, Thomas E, Snoswell CL, et al. Telehealth for global emergencies: Implications for coronavirus disease 2019 (COVID-19). J Telemed Telecare. 2020; 26(5): 309–313. DOI: 10.1177/1357633X20916567 32196391 PMC7140977

[12] Reynolds HN, Bander JJ. Options for tele-intensive care unit design: Centralized versus decentralized and other considerations. Crit Care Clin. 2015; 31(2): 335–350. DOI: 10.1016/j.ccc.2014.12.010 25814458

[13] Reynolds HN, Rogove H, Bander J, McCambridge M, Cowboy E, Niemeier M. A working lexicon for the tele-intensive care unit: We need to define tele-intensive care unit to grow and understand it. Telemed J E-Health Off J Am Telemed Assoc. 2011; 17(10): 773–783. DOI: 10.1089/tmj.2011.0045 22029748

[14] Ramnath VR, Ho L, Maggio LA, Khazeni N. Centralized monitoring and virtual consultant models of tele-ICU care: A systematic review. Telemed J E-Health Off J Am Telemed Assoc. 2014; 20(10): 936–961. DOI: 10.1089/tmj.2013.0352 25226571

[15] Ramnath VR, Khazeni N. Centralized monitoring and virtual consultant models of tele-ICU care: A side-by-side review. Telemed J E-Health Off J Am Telemed Assoc. 2014; 20(10): 962–971. DOI: 10.1089/tmj.2014.0024 25225795

[16] Carlson RW. Telemedicine in the ICU, An Issue of Critical Care Clinics, E-Book. Vol 31 Elsevier Health Sciences; 2015 DOI: 10.1016/j.ccc.2015.01.001 25814459

[17] Koenig M. Telemedicine in the ICU 2019 https://public.ebookcentral.proquest.com/choice/publicfullrecord.aspx?p=5771289 Accessed June 25, 2020. DOI: 10.1007/978-3-030-11569-2

[18] Project ECHO. COVID-19 Response. https://hsc.unm.edu/echo/institute-programs/covid-19-response/. Accessed November 5, 2020.

[19] Sisson P. On the Bleeding Edge of the COVID-19 Fight. San Diego Union-Tribune. 2020 https://www.sandiegouniontribune.com/news/health/story/2020-06-13/on-the-bleeding-edge-of-the-covid-19-fight. Accessed June 24, 2020.

[20] Compte O, Postlewaite A. Confidence-enhanced performance. Am Econ Rev. 2004; 94(5): 1536–1557. DOI: 10.1257/0002828043052204

[21] Gershengorn HB, Kocher R, Factor P. Management strategies to effect change in intensive care units: Lessons from the world of business. Part III. Effectively effecting and sustaining change. Ann Am Thorac Soc. 2014; 11(3): 454–457. DOI: 10.1513/AnnalsATS.201311-393AS 24601653

[22] Wiltsey Stirman S, Kimberly J, Cook N, Calloway A, Castro F, Charns M. The sustainability of new programs and innovations: A review of the empirical literature and recommendations for future research. Implement Sci. 2012; 7(1): 17 DOI: 10.1186/1748-5908-7-17 22417162 PMC3317864

[23] Lingard L, Espin S, Evans C, Hawryluck L. The rules of the game: Interprofessional collaboration on the intensive care unit teamound. Crit Care. 2004; 8(6): R403 DOI: 10.1186/cc2958 15566584 PMC1065058

[24] Manthous CA, Hollingshead AB. Team science and critical care. Am J Respir Crit Care Med. 2011; 184(1): 17–25. DOI: 10.1164/rccm.201101-0185CI 21471081

[25] Johnson EE, Sterba KR, Goodwin AJ, et al. Implementation of an academic-to-community hospital intensive care unit quality improvement program. Qualitative analysis of multilevel facilitators and barriers. Ann Am Thorac Soc. 2019; 16(7): 877–885. DOI: 10.1513/AnnalsATS.201810-735OC 30822096

[26] Berenson RA, Grossman JM, November EA. Does telemonitoring of patients—the eICU—improve intensive care?: A lack of hard data to answer the question argues for doing comparative effectiveness research on care delivery. Health Aff (Millwood). 2009; 28(Supplement 1): w937–w947. DOI: 10.1377/hlthaff.28.5.w937 19696068

[27] Kahn JM. Intensive care unit telemedicine: Promises and pitfalls. Arch Intern Med. 2011; 171(6). DOI: 10.1001/archinternmed.2011.23 21444841

[28] Alhazzani W, Møller MH, Arabi YM, et al. Surviving sepsis campaign: Guidelines on the management of critically ill adults with coronavirus disease 2019 (COVID-19). Crit Care Med. 2020; 48(6): e440–e469. DOI: 10.1097/CCM.0000000000004363 32224769 PMC7176264

[29] Google. [Google Maps Geography of Southern California region of USA and Northern Baja region of Mexico]. Published n.d. https://www.google.com/maps/place/San+Diego,+CA/@32.9363987,-116.7759926,9z/ data=!4m5!3m4!1s0x80d9530fad921e4b:0xd3a21fdfd15df79!8m2!3d32.715738!4d-117.1610838. Accessed July 13, 2020.

