# Supplementary material for: An In-Person and Telemedicine “Hybrid” System to Improve Cross-Border Critical Care in COVID-19
Source: Ann Glob Health. 2021 Jan 4;87(1):1. doi: 10.5334/aogh.3108 (PMC7792461; doi:10.5334/aogh.3108)
Supplement: Appendix 1. — Data fields in the cloud-based spreadsheet for Tele-ICU provider interventions and assessments. Providers indicated all interventions ordered and assessments made for each patient each day. Responses were binary (Yes/No), except in the “Other,” which was free text field. PBW = predicted body weight; MAP = mean arterial pressure; RASS = Richmond Agitation Sedation Scale; TOF = Train of Four. [file agh-87-1-3108-s1.pdf]

Appendix 1. Data fields in the cloud-based spreadsheet for Tele-ICU provider interventions and assessments. Providers indicated all interventions ordered and assessments made for each patient each day. Responses were binary (Yes/No), except in the "Other," which was free text field. PBW = predicted body weight; MAP = mean arterial pressure; RASS = Richmond Agitation Sedation Scale; TOF = Train of Four.

**Category of critical care management**	**Issues/problems for which specific interventions made to adhere with evidence-based guidelines**
Prone positioning	• Institution, timing, duration
Advanced ventilator management	• Ventilator dyssynchrony<br>• Excess driving pressure<br>• Excess plateau pressure/ lung compliance<br>• Presence of significant auto-PEEP<br>• Excess tidal volume (> 6 cc/kg PBW)<br>• Abnormal endotracheal tube positioning<br>• Lack of appropriate trials of liberation/weaning from mechanical ventilation
Hemodynamic management	• Vasopressor agent choice(s), titration to MAP goal, side effects/complication management
Fluid management	• Appropriate intravenous fluid choice(s), administration/titration goals, concentration of infusions<br>• Diuresis<br>• Timing and type of renal replacement therapy (e.g. hemodialysis)

Sedation/neuromuscular antagonist management	• Sedative and neuromuscular antagonist choice, titration to RASS/TOF goals, side effects/complication management
Nutrition and bowel motility	• Choice of feeding type, formula, route, dosing, side effect/complication management<br>• Choice of bowel motility agent(s), side effect/complication management
Venous thromboembolism and stress ulcer prophylaxis	• Choice of agent(s), route, dosing, side effect/complication management
