# Supplementary material for: An In-Person and Telemedicine “Hybrid” System to Improve Cross-Border Critical Care in COVID-19
Source: Ann Glob Health. 2021 Jan 4;87(1):1. doi: 10.5334/aogh.3108 (PMC7792461; doi:10.5334/aogh.3108)
Supplement: Appendix 2. — Survey questions as part of staff perception evaluation. RN = registered nurse; RT = respiratory therapist. [file agh-87-1-3108-s2.pdf]

Appendix 2. Survey questions as part of staff perception evaluation. RN = registered nurse; RT = respiratory therapist.

Survey Question	Response options
Before Tele-ICU started at ECRMC (April 6, 2020), how confident did you feel in managing critically ill NON-COVID-19+ patients in ICU?	• Not confident at all<br>• Slightly confident<br>• Moderately confident<br>• Very confident<br>• 100% confident, didn't need any help
Before Tele-ICU started at ECRMC (April 6, 2020), how confident did you feel in managing critically ill COVID-19+ patients in ICU?	• Not confident at all<br>• Slightly confident<br>• Moderately confident<br>• Very confident<br>• 100% confident, didn't need any help
With Tele-ICU at ECRMC, how confident do you feel in managing critically ill NON-COVID-19+ patients in ICU now?	• Much less confident or not confident at all<br>• Slightly less confident<br>• Neutral<br>• Slightly more confident<br>• Definitely more confident or 100% confident
With Tele-ICU at ECRMC, how confident do you feel in managing critically ill COVID-19+ patients in ICU now?	• Much less confident or not confident at all<br>• Slightly less confident<br>• Neutral

	• Slightly more confident<br>• Definitely more confident or 100% confident
What percentage of ECRMC ICU patients do you think received evidence-based practice of critical care BEFORE Tele-ICU started?	0–100%
What percentage of ECRMC ICU patients do you think receive evidence-based practice of critical care NOW?	0–100%
What do you think is the highest value of the Tele-ICU service?	• Critical care advice for mechanically ventilated (i.e. intubated/BiPAP) patients in ICU<br>• Critical care advice for non-ventilated patients in ICU<br>• Ability to ask medical questions to a critical care specialist<br>• Facilitated patient transfer to other hospitals (e.g. UCSDHS)<br>• Access to potential ECMO for patients through UCSDHS<br>• Other
Which of the following needs improvement to make Tele-ICU better?	• Better communications by UCSDHS tele-intensivist about orders/plan with ECRMC

	RN/RT<br>• More orders entered by UCSDHS tele-intensivist<br>• More patients seen by UCSDHS tele-intensivist<br>• UCSDHS tele-intensivist notes can be more completely written<br>• More coverage hours for UCSDHS tele-intensivist to manage ICU patients<br>• Consistency of plan between different tele-intensivists<br>• Consistency of plan between tele-intensivists and onsite physicians (e.g. ECRMC hospitalists)<br>• Nothing – everything is working well
How many hours per day would be preferred for UCSDHS tele-intensivist service coverage (4, 8, 12, 24 hours)?	• 4 hours (no change)<br>• 8 hours<br>• 12 hours<br>• 24 hours
