# Supplementary material for: An In-Person and Telemedicine “Hybrid” System to Improve Cross-Border Critical Care in COVID-19
Source: Ann Glob Health. 2021 Jan 4;87(1):1. doi: 10.5334/aogh.3108 (PMC7792461; doi:10.5334/aogh.3108)
Supplement: Appendix 3. — Assessed factors upon initial assessment. HGT = Tijuana General Hospital, HMG = Mexicali General Hospital, ECRMC = El Centro Regional Medical Center, HME = Heat and Moisture Exchangers, EMR = Electronic Medical Record, EUA = Emergency Use Authorization. [file agh-87-1-3108-s3.pdf]

Appendix 3. Assessed factors upon initial assessment. HGT = Tijuana General Hospital, HMG = Mexicali General Hospital, ECRMC = El Centro Regional Medical Center, HME = Heat and Moisture Exchangers, EMR = Electronic Medical Record, EUA = Emergency Use Authorization.

Feature		HGT	HGM	ECRMC
**Plant structure**	Capacity of ICU beds	14	64, but capacity up to 105 if staffing permits	16, with plan for expansion to 28
	Accessory (overflow) units active for COVID	✓	✓	✓
	Antechamber for donning and doffing	✓	✓	Variable based on patient room
	Conference room available for Tele-ICU	✓	✓	N/A
**Infection**	Individual	Not present	Not present	✓

**control methods**	patient rooms/ isolation			
	COVID cohort units	✓	✓	✓
**Equipment**	Ventilators	✓	✓	✓
	HME	Originally more needed, but now near sufficient	Present	Present
	Monitors for vented patients	More needed	Present	Present
	Portable ultrasound devices for bedside evaluation	More needed	Present	Present
	Computers available	More needed	Present	Present
	EMR	Not present	Present	Present
	Renal	Hemodialysis only	Hemodialysis	Hemodialysis only

	replacement therapies	(no CRRT; potentially available at patient cost)	only (no CRRT)	(no CRRT)
	EEG	Available	Not available	Not available
	MRI	Not available	Not available	Available, but not for COVID patients
	CT scanner	Not presently available	Available	Available
	Microbiology lab	Not presently available	Not presently available	Available
**Personnel**	Respiratory therapists	Several available for equipment maintenance, not patient care	Not available	✓
	Critical Care nurses	More needed	More needed	✓
	Critical Care-trained intensivists	More needed	More needed	Not present/variable
	Hemodialysis nurses	More needed	More needed	More needed

	In house anesthesiology	Not available	Not available	Not available
**Supplies**	Medications	• Remdesivir not available in Mexico<br>• Fentanyl, morphine or opiates unavailable (only buprenorphine)<br>• Dexmedetomidine available<br>• Tocilizumab, baricitinib available (off-label)<br>• Convalescent plasma possible but not yet used	• Remdesivir not available in Mexico<br>• Convalescent plasma not available<br>• Dexmedetomidine available but low quantities<br>• Tocilizumab, baricitinib available only at patient cost (off-label)	• Dexmedetomidine originally not available but became readily available after Tele-ICU program launch<br>• Remdesivir available, but low quantities<br>• Tocilizumab, baricitinib, convalescent plasma available (as part of study trials and EUA)
